# A student-centered approach for developing active learning: the construction of physical models as a teaching tool in medical physiology

**DOI:** 10.1186/1472-6920-14-189

**Published:** 2014-09-15

**Authors:** Flávio Moura Rezende-Filho, Lucas José Sá da Fonseca, Valéria Nunes-Souza, Glaucevane da Silva Guedes, Luiza Antas Rabelo

**Affiliations:** Laboratório de Reatividade Cardiovascular, Setor de Fisiologia e Farmacologia, Instituto de Ciências Biológicas e da Saúde, Universidade Federal de Alagoas, Av. Lourival de Melo Mota S/N. Bairro Tabuleiro dos Martins, Maceió, 57072-900 Alagoas Brazil; Rede Nordeste de Biotecnologia (RENORBIO), Ponto Focal Maceió, Maceió, Alagoas Brazil; Instituto Nacional de Ciência e Tecnologia em NanoBiofarmacêutica (N-BIOFAR), Belo Horizonte, Brazil; Max-Delbrück-Center for Molecular Medicine, Berlin, Germany; Faculdade de Nutrição (FANUT), Universidade Federal de Alagoas, Maceió, Alagoas Brazil

**Keywords:** Active learning, Meaningful learning, Teaching-learning methodologies, Medical education

## Abstract

**Background:**

Teaching physiology, a complex and constantly evolving subject, is not a simple task. A considerable body of knowledge about cognitive processes and teaching and learning methods has accumulated over the years, helping teachers to determine the most efficient way to teach, and highlighting student’s active participation as a means to improve learning outcomes. In this context, this paper describes and qualitatively analyzes an experience of a student-centered teaching-learning methodology based on the construction of physiological-physical models, focusing on their possible application in the practice of teaching physiology.

**Methods:**

After having Physiology classes and revising the literature, students, divided in small groups, built physiological-physical models predominantly using low-cost materials, for studying different topics in Physiology. Groups were followed by monitors and guided by teachers during the whole process, finally presenting the results in a Symposium on Integrative Physiology.

**Results:**

Along the proposed activities, students were capable of efficiently creating physiological-physical models (118 in total) highly representative of different physiological processes. The implementation of the proposal indicated that students successfully achieved active learning and meaningful learning in Physiology while addressing multiple learning styles.

**Conclusion:**

The proposed method has proved to be an attractive, accessible and relatively simple approach to facilitate the physiology teaching-learning process, while facing difficulties imposed by recent requirements, especially those relating to the use of experimental animals and professional training guidelines. Finally, students’ active participation in the production of knowledge may result in a holistic education, and possibly, better professional practices.

## Background

Taking into account that innovation in medical education is not an easy task [1], a considerable body of knowledge about cognitive processes and methods of teaching and learning has accumulated in recent decades, helping teachers to determine the most efficient way to teach. Scientific evidence has shown that active student participation facilitates the assimilation and consolidation of new knowledge, and improves learning outcomes [2–5]. In addition, literature favors the concept that information should be provided simultaneously in multisensory modalities, since a considerable amount of data indicates that the most complete and successful strategy for teaching physiology to a diverse group of students consists in offering information in a manner that addresses multiple learning styles [6–11], satisfying visual, aural and kinesthetic learners [2, 12–15].

Some of these methodologies are particularly suitable when physiology teacher’s focus is on fostering the rational use of experimental animals [16, 17] and promoting the acquisition of skills and competencies such as communication, critical thinking and teamwork. In this sense, models are frequently used to explain complex ideas, since they incite logical reasoning and creativity, enabling students to develop conceptual or qualitative representations of the subject matter, directing them towards meaningful learning [2, 3, 8, 12, 14, 18, 19].

In Brazil, the intrinsic difficulties in implementing more efficient ways to teach physiology were recently augmented by new demands of the National Curriculum Guidelines for health professionals, and by ethical restrictions imposed on the use of experimental animals [16, 17]. To meet these new requirements, our institution implemented a Pedagogical Policy Plan centered on Problem-Based Learning (PBL) for the undergraduate medical course [3, 11, 20, 21], to which our department of Physiology had to adapt. Since the use of manipulative activities does not require animals and has previously shown good learning outcomes [7, 14], it has emerged as a promising alternative based on which we have developed a teaching and learning methodology.

This paper describes the process of teaching and learning physiology through the construction and presentation of physiological-physical models (PPMs), focusing on their potential use in the practice of teaching physiology. What makes our experience different is that students represent the core of the process, not passively receiving a proposal for constructing a PPM, but creating it instead, and that all the PPMs are presented in a Symposium on Integrative Physiology (SIP), an academic event especially created for this purpose.

## Results

After applying the proposed methodology, students were able to develop PPMs that effectively represented different types of physiological processes. The closure of activities took place in an event designed specifically for the integration of knowledge in physiology (the SIP) through the presentation of 8–23 different Physiology models in each edition. In fact, it was a consensus among the evaluators that all the PPMs presented up to the last SIP met the evaluation criteria of uniqueness, creativity and representativeness of the physiological topic in question, and most of the models were rated very good or excellent (scores of 8 or higher on a scale of 0 to 10).

**Figure 1:**
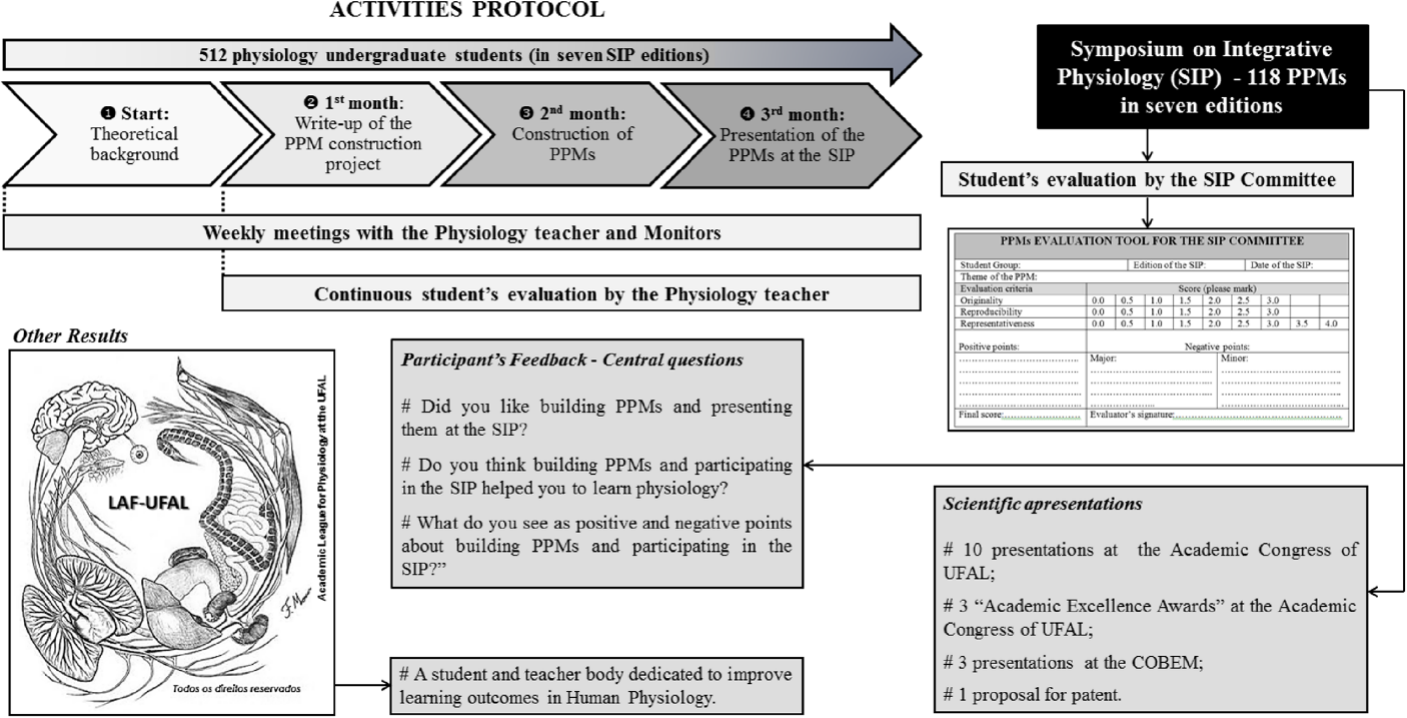
**Workflow representing the sequential steps that culminated with the presentations at the Symposium on Integrative Physiology.** COBEM, Brazilian Congress of Medical Education (“Congresso Brasileiro de Educação Médica”); LAF, Academic League for Physiology (“Liga Acadêmica de Fisiologia- LAF/UFAL”); SIP, Symposium on Integrative Physiology; PPMs, physiological-physical models; UFAL, Federal University of Alagoas (“Universidade Federal de Alagoas”).

**Figure 2:**
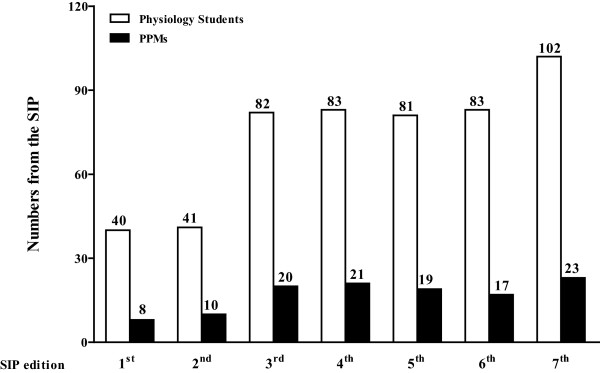
**Number of participants and the respective number of physiological-physical models (PPMs).** Numbers correspond to the amount of PPMs on each edition of the Symposium on Integrative Physiology (SIP). Data are presented as absolute numbers.

**Figure 3:**
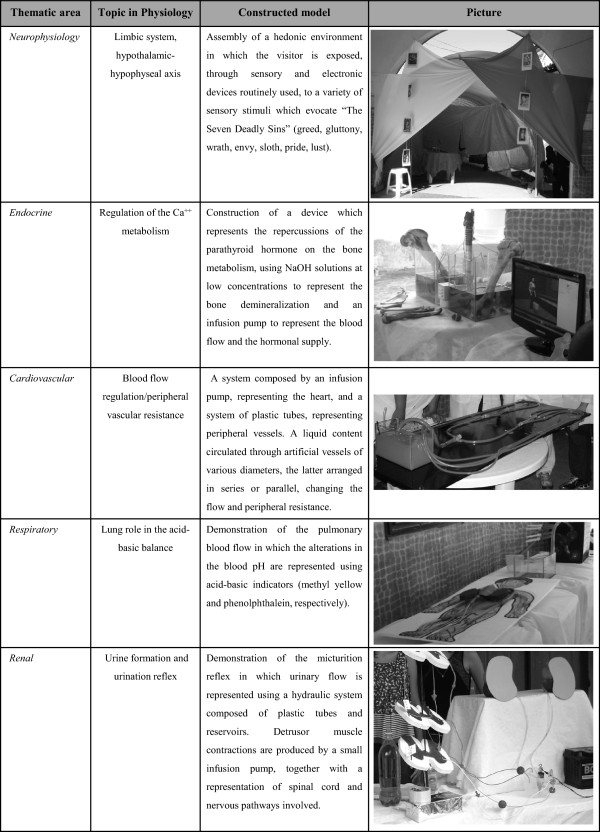
**Physiological-physical models (PPMs) built by students for the presentation at the Symposium on Integrative Physiology.** The PPMs are grouped by specific areas of Physiology. One model is numbered for each considered topic, being representative of the total production in the Symposium on Integrative Physiology (SIP).

So far, our students have created 118 PPMs (Figures 1 and 2) (11 addressing renal physiology, 12 related to respiratory mechanisms, 16 concerning endocrinology and metabolism, 32 representing different aspects of the cardiovascular system and 47 related to neurophysiology, with 105 of them unpublished); 10 presentations at the Academic Congress of UFAL (2009), with three Academic Excellence Awards received during the aforesaid congress; three presentations at the 49^th^ Brazilian Congress of Medical Education (COBEM), and one proposal for patent (Figure 1). The SIP has already held seven editions. One model from each area of Physiology considered in the SIP is described in Figure 3. During the semesters considered, 15.24% of the medical students participated in the SIP, which is a representative percentage, once all of those who were studying Physiology at that time (512 in a total of 3.360 medical students at UFAL in seven semesters) undergone the practical activity. The percentage constitutes a significant number taking into account that the scope of the study was to reach as many Physiology students as possible, and this aim was achieved successfully.

Concerning the participant’s feedback, some of the comments received in response to the questionnaire were selected according to the most frequently cited ones, and are given in Table 1.

More importantly, some students were so gratified with the use of this teaching-learning method that they visited our department frequently to discuss it, and this interest resulted in the creation of the Academic League for Physiology at the Federal University of Alagoas (LAF-UFAL), a student and teacher body dedicated to improve learning outcomes in Human Physiology (Figure 1).

**Table 1:** **Central ideas present in responses to the feedback questionnaire and their respective representative comments**

*Central ideas*	*Representative comments*
PPMs construction and the SIP helped students to learn.	*“…it is not only the model, the final product, that teaches, but the whole process by which the students undergo till get to finish the complete model…”.*
The strategy fostered the development of skills, such as teamwork.	*“I underwent two symposiums. They extremely encourage creativity, allowing to explore the domain of the theoretical content, and proportion an improvement in the teamwork capacity”.*
PPMs and the SIP resulted in secondary benefits, such as possible publications.	*“…the presentation to the public, which shows itself extremely interesting, and for the secondary benefits, once with my two models I won an Academic Excellence Award and got a possible publication”.*
Students liked presenting models in the SIP.	*“It is a really challenging proposal, to create a model of practical class. The results are generally great and the day of the presentation is very interesting”.*
PPMs and the SIP were a heavy workload during the semester	*“…these symposiums became a heavy burden considering the tight timetables of our course, though the symposium is dynamic, interesting and very instructive”.*
During the SIP, there was not enough time for all students to see the presentation of other groups.	*“…I studied a lot and learned in an efficient way at least the issue I was presenting… I can’t say the same about the other presented works, because we had very little time to visit the other groups”.*

Individual students’ responses about their experiences during their participation in the Symposium on Integrative Physiology.

Each reported fragment represents part of the descriptions made by the students while responding to the questionnaire about the activities performed during the Symposium on Integrative Physiology (SIP). The results presented were selected from different semesters which underwent the proposed protocol.

Table 2 describes some of our observations about active learning and meaningful learning during the application of the proposed methodology. The information provided served to address different learning styles. We also believe that our protocol of activities encouraged the development or enhancement of skills and competencies relevant to health professionals, such as critical thinking, teamwork, and communication.

**Table 2:** **Students’ activities and their potential effects on the teaching-learning process**

Activities	Potential effect on the teaching-learning process
# Interacting with other students and teachers by discussing ideas and concepts related to physiology and to the construction of the models.	Meaningful Learning
# Assembling cheap inert recycled materials and objects with no physiological significance to create a system or model that represents a physiological process.	
# Articulating explanations about the topic in human physiology that the model is about and how it represents the physiological process in question.	
# Answering other students and teachers’ questions about the model and the physiological process it exemplifies.	
# Being responsible for the design, preparation and presentation of a PPM that represents a specific physiological process.	Active Learning
# Engaging in the activities of model building, project writing, presentation of the model, and interacting with the creators of other PPMs at the SIP event.	
# Exploring their own attitudes, values and beliefs about the teaching-learning process when confronted by those of classmates during the group work.	
# Constantly reflecting on ideas and concepts in order to improve physiological models or overcome failures in their operation.	

The information is organized according to the different activities numbered along the protocol of activities.

## Discussion

This article describes a teaching and learning active methodology based on the use of physical models as tools in medical physiology. The fact that, at the end of the process, students were capable of successfully creating their own PPMs with great representativeness of physiological phenomena presents itself as the main finding of the reported experience.

Concerning the effectiveness of the methodology described when it comes to the models used as learning tools, the integrative character of the proposal allowed to transpose physiological concepts from theory to an immediate and practical application. As shown in Figure 3, the level of difficulty for the construction of each model was directly related to the conceptual design and the materials used for concretizing the idea. Students were stimulated to use, whenever possible, materials easily available, such as recyclable ones. At specific circumstances, however, special components (e.g. a car battery or electronic systems) were required for properly achieving their goals, bringing a higher level of difficulty for finalizing the constructions.

Despite such variations in the degree of complexity for building the PPMs, our results indicate that, under favorable conditions, students are perfectly capable of designing and building models based on Physiology-related topics on their own, suggesting that the method could be reproducible in other schools. As for the PPMs themselves, all of them were built predominantly using recycled materials, which are cheap and readily available almost everywhere in the world. The use of such tool can be considered a valuable alternative, in view of the ethical and legal barriers imposed on animal experimentation in Brazil and elsewhere. Although sophisticated industrial simulators are available for use in practical Physiology classes, many medical schools in developing countries cannot afford them; hence, their students would benefit from using such handmade models. The PPMs developed by our undergraduates and presented at the SIP were considered highly representative of their respective physiological topics, encouraging discussions about the theme and promoting student-student and student-teacher interactions. This could be attributed to the interactive nature of the models, whose colors, movements, sounds and even smells and tastes captivated the audience, creating an enjoyable atmosphere of excitement and passion for learning during the SIP, as observed in experiences described by other groups [13, 22].

The feedback from students about the approach used was excellent, with an overwhelming majority of students reporting positive results and that the creation of the PPMs helped them learn. In addition, students were also asked to give their verbal feedback, and all the groups stated that the protocol in which they participated helped them to learn and fostered the development of certain skills. The challenge of creating a new, original teaching model of a physiological process is an unusual situation for undergraduate Physiology students, which leads them to find a solution not available in articles or textbooks, thus firing their creativity.

Another important point is the fact that the teamwork was supported from the initial stages of our methodology. In this regard, the fact that students were always working in groups is of particular note, since it is already described that individuals are likely to learn more effectively when they learn with others than when working alone [6, 7]. In our study, teachers and PMs observed something similar during group discussions in weekly meetings (each discussion section lasting 15 minutes on average), when some questions were answered by the students themselves, despite the presence of the teacher and/or monitor in the meeting. Hence, the present strategy promoted ongoing teamwork, translating into better learning outcomes and enhancing creative thinking skills.

In consonance with these observations, since teamwork is not possible in the absence of communication [23, 24], the students had to express their ideas accurately and understandably to their classmates, PMs and teachers. Thus, students had the opportunity to improve their communication skills. Considering the fact that creating a practical model necessarily requires an in-depth review of the literature, the proposal has proved to be an important way of consolidating their knowledge about the subject matter, which is unquestionably a crucial step in “learning how to learn.” In this sense, the activity takes on an educational perspective, which is another critical pillar of the method. It encourages the student from the start of the course, and may later be reflected in a deeper interest in the subject, making it easier to understand the subsequent subjects that depend on Physiology [6], leading to better outcomes in these specific areas. The results suggest that it is feasible to implement a student-centered teaching-learning method based on the construction of PPMs at little cost. Also, the methodology presented herein is to a certain extent innovative by including elements of other teaching strategies, such as PBL, articulating explanations and group work [2–4, 8, 12, 14, 18, 19].

Since the integrated curriculum has recently emerged and presents itself as a current trend in medical education, one could wonder where the proposed methodology would fit, considering the strict timetables available for Physiology classes. In this regard, once the proposal described herein is based on an integrative perspective, we do believe it could be applied not only to the field of Physiology, but also to combine different disciplines in an integrated curriculum. Furthermore, counting on the students’ commitment in performing the activities out of the classroom, at specific moments previously defined (such as observed in the SIP), it would be possible to conduct the methodology smoothly, provided that it does not compromise the curricular activities.

The models envisioned by the groups were designed and tested several times and most of them had several trial versions before reaching the final version. Thus, it becomes plausible to suggest that when students were building a particular model, giving it a physical body and testing it, they were actually testing their mental models of the Physiology topic on which they were working [2, 12, 19]. There is also a second stage in which active learning takes place, that is, during the SIP, when the groups had the opportunity to shift their focus of attention from their own models to those of other groups, concentrating on them and thus exploring their operation. The occurrence of active learning during this type of activity is seen in many teaching-learning experiences previously reported [3, 5, 7]. In view of these observations, it is plausible to postulate that active learning occurred during this study (Table 2). Furthermore, our observations of student work and activities led us to conclude that the construction of a Physiology-related PPM potentially requires the elaboration of diverse mental representations linked to the Physiology topic under study.

Still considering the presentations at the SIP, it should be kept in mind that it was a moment when each student was required to express himself about his group’s model, the physiological process that the model purportedly represented, and how the model reproduced the physiological process (Table 2). In addition, the possibility of interaction with the PPMs during the SIP enabled students to reinforce physiological concepts, since most of the presentations required the presenter or the spectator to touch or handle the physical models, ultimately addressing multiple learning styles. We believe this was a critical component of the method applied, since the most complete and successful strategy for teaching physiology to a diverse group of students consists of offering information in a manner that addresses multiple learning styles [3, 8, 9]. In this sense, collectively, our findings are consistent with those of other experiences in the use of models to improve learning [2, 8, 12, 14, 18, 19], which have reported better outcomes than those achieved with traditional teaching methodologies [7, 14].

## Conclusion

In summary, our teaching-learning methodology produced good learning outcomes. The proposal presented herein has proved to be an attractive and useful approach to facilitate the Physiology teaching-learning process. Furthermore, the method is an accessible and relatively easy way to foster meaningful learning and active learning while concomitantly addressing multiple learning styles.

While assessing this proposal as a whole, some aspects should be considered, as observed in other suggested implementations [5, 15]. First, the population of students that take Physiology courses is likewise very diverse and represents many different ages, and cultural and educational backgrounds. In this sense, applying the protocol of activities in other institutions may result in considerably different outcomes. Second, we only performed evaluations during the development of activities. A longer follow-up is necessary to determine whether acquired knowledge was long-lasting or not. Our group is currently addressing this issue. Finally, the teaching-learning strategy presented herein could not be compared with more traditional approaches. Although literature indicates effective learning is more likely to occur in a scenario similar to the one we settled, solid evidence favoring our strategy could only be provided by comparative analysis of learning outcomes.

### Perspectives

Having successfully achieved and implemented the proposed approach, we intend to extend this methodology in the near future to other health sciences courses by inviting other teachers to become regular participants. This would allow us to establish an integrated symposium for all the biological and biomedical courses in our University. Based on this invitation, a pilot project was implemented at our institution in previous semesters, with the participation of the Nutrition course. By this means, we intend to ensure that other undergraduate health sciences courses will benefit from our approach, sharing new experiences and learning together, as should be the case in professional environments after graduation. Furthermore, a suggestion for another feedback tool is already being elaborated by our group.

## Methods

### Conception of the teaching and learning strategy

The development of this teaching and learning strategy was guided by our country’s curriculum guidelines for health professional training [20] and our institution’s Pedagogical Policy Plan for the undergraduate medical course [11]. The methodological platform used for developing this strategy was also supported by the work of other authors in the field of Physiology education [6, 25], which was based on teamwork, sharing acquired knowledge among groups [6], ultimately developing critical-thinking from basic concepts to their applicability in clinical practice [25], and our group’s experiences. The strategy proposed was based on the following characteristics: *a)* no requirement for the use of experimental animals; *b)* encouragement of active learning; *c)* promotion of meaningful learning; *d)* meet the needs of as many students as possible in terms of different learning styles; and *e)* stimulate the development of skills and competencies essential for health professionals.

During the whole semesters, activities in which students engaged were carefully documented on activities logs, filled by the Physiology teacher and/or Physiology Monitors (PMs). At the end of each semester, data were descriptively analyzed, in search for any signs of active learning and meaningful learning [9, 10]. Figure 1 summarizes the steps of the proposed teaching method.

### Participants

512 undergraduate students who participated in this proposal were all studying Human Physiology. In order to organize the implementation of the proposal, a Committee for the SIP was defined. The latter consisted of four students who participated in the first edition, and became PMs in the following editions, after receiving specific training in the teaching and learning methodology. Four guest teachers were invited to evaluated students and their work, together with the Physiology teacher.

The activities conducted with undergraduate students of Human Physiology (medical course, Federal University of Alagoas) in the period between 2007 and 2010 were exempted from the submission to the Institutional Ethical Committee. The exemption is justified because all the methodological procedures applied along the proposal, the latter based on the development of PPMS, are in accordance with the institution’s Pedagogical Policy Plan (and, consequently, with its respective curriculum) for the undergraduate medical course.

### Activities protocol

The approach started in the classroom, where the aforementioned teaching-learning methodology was proposed to the students as an assessment criterion for the discipline of Physiology. Then, students were divided into groups of 3 to 5 individuals. Next, in order to ensure that students’ active participation would be present since the initial steps of the proposal, each group was assigned or suggested a topic from different areas of Physiology, depending on the module they were studying at the time, so that groups could feel free to select the theme/subtheme they would work with. Considering all the activities through which each group underwent along the whole semester, students used approximately 16 hours to complete their models, from the conception until the effective construction of them. The protocol of activities designed for this experiment was applied as follows (Figure 1):

1. **Theoretical background:** Students took classical classes in Physiology and received continuous guidance about the construction of the PPMs. Two-hour meetings with teachers, PMs and postgraduate students were scheduled once a week (Step 1, Figure 1).
2. **Write-up of the PPM construction project:** During the write-up of their PPM projects, the students had to make a brief review of the literature to present their models theoretically and explain the physiological mechanisms involved, with this step taking approximately two hours (Step 2, Figure 1).
3. **Construction of PPMs:** Students had to work with materials of non-animal origin to build their models. They were allowed to interact with other sectors of the university and with other institutions, according to the study field of the PPM. At this point, the groups were also taking scientific methodology classes about how to prepare posters to be presented together with the PPMs. After the groups concluded their projects, they wrote an initial abstract which was sent to the SIP Committee (Step 3, Figure 1). Depending on the complexity of the model designed, the time required varied from two to twelve hours, according to the students’ feedbacks.
4. **Presentation of the PPMs at the SIP:** The groups presented their models to all visitants. Each group had 20 to 30 minutes to present their PPM, explain and discuss its physiological concepts, and answer questions (Step 4, Figure 1).

### Student’s evaluation

For evaluating the PPMs, a scoring system ranging from 0 to 10 points was created according to three aspects: 1) Originality, depending whether similar apparatus were previously described, and how similar they were to the model under evaluation; 2) Reproducibility, depending on how cheap and available the components of the model were and how simple was its construction; 3) Representativeness, related to the capacity to represent a physiological process, explain and transmit the concepts involved. The assessment of the models was coupled with the ones of the oral presentation and posters, as well as the answers to evaluators’ questions (Figure 1).

All authors took responsibility for analyzing the data. Initially, the models which received higher scores after being presented in the SIP were selected as potential standards to represent the topics exposed at the end of activities. Finally, for choosing the models shown in Table 1, the authors had their opinions summarized by voting.

### Participant’s feedback

A previously designed questionnaire was sent to every participant of this proposal. It was composed of three questions (See Figure 1). The answers obtained were categorized and had their central ideas extracted. Some fragments of the comments are presented in Table 1.

## Abbreviations

PPMs: Physiological-physical models
PBL: Problem-based learning
SIP: Symposium on integrative physiology
PMs: Physiology monitors
COBEM: Brazilian congress of medical education
LAF-UFAL: Academic league for physiology at the Federal University of Alagoas.
